# Additional Selection for Insecticide Resistance in Urban Malaria Vectors: DDT Resistance in *Anopheles arabiensis* from Bobo-Dioulasso, Burkina Faso

**DOI:** 10.1371/journal.pone.0045995

**Published:** 2012-09-25

**Authors:** Christopher M. Jones, Hyacinthe K. Toé, Antoine Sanou, Moussa Namountougou, Angela Hughes, Abdoulaye Diabaté, Roch Dabiré, Frederic Simard, Hilary Ranson

**Affiliations:** 1 Vector Group, Liverpool School of Tropical Medicine, Liverpool, Merseyside, United Kingdom; 2 Institut de Recherche en Sciences de la Santé/Centre Muraz, Bobo-Dioulasso, Burkina Faso; 3 Centre National de Recherche et de la Formation sur Paludisme, Ouagadougou, Burkina Faso; 4 Maladies Infectieuses et Vecteurs : Ecologie, Génétique, Evolution et Contrôle, Institut de Recherche pour le Développement, Montpellier, France; Instituto de Higiene e Medicina Tropical, Portugal

## Abstract

In the city of Bobo-Dioulasso in Burkina Faso, *Anopheles arabiensis* has superseded *Anopheles gambiae s.s.* as the major malaria vector and the larvae are found in highly polluted habitats normally considered unsuitable for Anopheles mosquitoes. Here we show that *An. gambiae s.l.* adults emerging from a highly polluted site in the city centre (Dioulassoba) have a high prevalence of DDT resistance (percentage mortality after exposure to diagnostic dose = 65.8% in the dry season and 70.4% in the rainy season, respectively). An investigation into the mechanisms responsible found an unexpectedly high frequency of the *1014S kdr* mutation (allele frequency = 0.4), which is found at very low frequencies in *An. arabiensis* in the surrounding rural areas, and an increase in transcript levels of several detoxification genes, notably from the glutathione transferase and cytochrome P450 gene families. A number of ABC transporter genes were also expressed at elevated levels in the DDT resistant *An. arabiensis*. Unplanned urbanisation provides numerous breeding grounds for mosquitoes. The finding that Anopheles mosquitoes adapted to these urban breeding sites have a high prevalence of insecticide resistance has important implications for our understanding of the selective forces responsible for the rapid spread of insecticide resistant populations of malaria vectors in Africa.

## Introduction

Almost half of the human population in Sub-Saharan Africa are projected to live in urban areas by 2030 [1]. Although urbanisation is typically thought to reduce malaria transmission [2], the rapid and unplanned growth of towns and cities is generally associated with inferior housing, poor sanitation, and increased pollution, all of which could impact on the distribution and abundance of mosquito vectors [3], [4]. An evidence-based approach to understanding the ecology of urban malaria vectors for better control is therefore required.

*Anopheles arabiensis* is one of five mosquito species known to transmit malaria in African urban settings [3]. This member of the *Anopheles gambiae* species complex has a wide geographical range extending across the continent and is associated with drier climates and extensive land clearance [5]. Deforestation and urbanisation have created an arid environment into which *An. arabiensis* has invaded and adapted [6] and this species is the dominant malaria vector in several African cities [7]–[10]. The invasion of *An. arabiensis* into these ‘urban islands’ may be attributable to local adaptation to atypical breeding sites such as polluted water pools or ditches [8], [11]–[13].

High coverage with insecticide-based interventions such as long-lasting insecticide treated nets (LLINs) and indoor residual spraying (IRS) has made a major contribution to reducing the malaria burden in Africa [14]. Insecticide resistance represents a serious threat to this progress particularly in light of ambitious plans to further scale-up coverage with these interventions [15]. Pyrethroids remain the only compound licensed for use on LLINs while the limited arsenal of available insecticides has led to a resurgence in the use of DDT for IRS [16]. Reports of pyrethroid and DDT resistance in *Anopheles* are widespread throughout Africa [17]–[19]. Two mechanisms have been conclusively shown to cause insecticide resistance in mosquitoes: i) mutations in the target-site protein of the insecticide and ii) increased detoxification of the insecticide by metabolic enzymes (e.g. P450 monooxygenases, glutathione-S-transerferases (GSTs) and carboxyesterases). DDT and the pyrethroid insecticides target the voltage-gated sodium channels (VGSC) in the nerve membranes of insects. Cross-resistance to these compounds in *Anopheles* is associated with two variant knockdown resistance (otherwise known as *kdr*) mutations at position 1014 in the VGSC; a leucine to phenylalanine (*L1014F*) and a leucine to serine (*L1014S*) substitution [20], [21]. These *kdr* alleles are widely distributed in *An. gambiae s.l.* throughout Africa and have been subject to strong positive selective sweeps [22], [23]. Despite the presence of both *1014F* and *1014S* in West African *An. arabiensis* [24], [25], allele frequencies are much lower than those reported in M- and S-molecular forms of *An. gambiae s.s.* [18], [26].

Gene expression profiling using targeted microarrays has proven a powerful experimental tool for identifying candidate genes associated with insecticide resistance [27]–[29]. In *An. gambiae*, several detoxification genes have been identified that are over-expressed in multiple insecticide resistant populations and the ability of several candidate cytochrome P450s and glutathione transferases (GSTs) to metabolise DDT and/or pyrethroids confirmed [29]–[32]. Less attention has been paid to other putative resistance mechanisms but the development of robust whole-genome microarrays [33] permits a much broader approach to studying this area.

The city of Bobo-Dioulasso lies within a large cotton belt in the south-west of Burkina Faso. *An. gambiae s.s.* collected from central Bobo-Dioulasso previously displayed reduced susceptibility to pyrethroids and DDT [34]. The vector dynamics in this area have, however, changed over the past decade with *An. arabiensis* superseding *An. gambiae s.s.* as the main member of the *An. gambiae* complex [35]. In this paper, we describe a DDT resistant population of *An. arabiensis* from a polluted breeding site in Bobo-Dioulasso and explore the genetic basis of this resistance. The implications for understanding the selective pressures on insecticide resistance in urban malaria vectors are discussed.

## Materials and Methods

### Study Site and Mosquito Collections

Third and fourth stage *Anopheles* larvae were collected from Dioulassoba (11°10′42′′N; 4°17′35′′W), an urban district in the centre of Bobo-Dioulasso, the second largest city of Burkina Faso. This district is crossed by a permanent stream which is intermittently surrounded by vegetable cropping. The stream is polluted with domestic waste and organic refuge (e.g. empty containers and daily water-waste). Small puddles along the stream and the surrounding borders constitute permanent breeding sites for both *Anopheles* and *Culex* larvae. *Anopheles* larvae were collected using a standard dipping method in August 2009 and January 2010, during the rainy and dry season respectively. Larvae were reared to adults at the insectaries of IRSS/Centre Muraz and blood fed to generate the test population which was then used in bioassays for resistance to DDT.

### Ethics Statement

Dioulassoba has been a sentinel site of IRSS/Centre Muraz research team since the 1980s and, therefore, no specific work permits were required to work in this area. The stream is not privately owned and the field study did not involve protected or endangered species in any way.

### DDT Susceptibility Bioassays

Bioassays were conducted on adult mosquitoes emerging from larvae collected in the dry and wet seasons. Three to five day old adult females *An. gambiae s.l.* were exposed for one-hour to papers impregnated with DDT (4%) in World Health Organisation (WHO) susceptibility tests [36]. Mosquito mortality was recorded 24 hours later. Control assays were performed throughout the experiment with a minimum of 25 mosquitoes exposed to non-insecticide treated papers. Mosquitoes were left in holding tubes for a further 24 hours (48 hours post-exposure) to minimise the effect of induction on gene expression from insecticide exposure. Any mosquitoes which died during this period were not considered for molecular analysis. Between one and two legs were removed from all survivors (exposed and non-exposed) and placed on silica gel. The remainder of the insect was preserved in RNAlater (Sigma) for microarray experiments.

### Species Identification and kdr Genotyping

Genomic DNA (gDNA) was extracted from the legs of adult female *An. gambiae s.l.* by heating to 95°C for 30 minutes in 20 µl of 1× PCR buffer (Promega). This was subsequently used to distinguish between *An. arabiensis* and *An. gambiae s.s.* following the method described by Scott *et al.* [37].

The frequency of the *1014F* and *1014S kdr* alleles in *An. arabiensis* exposed and non-exposed to DDT was determined using the TaqMan® PCR assays described in Bass *et al*. [38]. TaqMan® PCR reactions were performed on the Agilent MX3005P qPCR system. Hardy-Weinberg equilibrium analysis was performed following Rodriguez *et al*. [39].

### Microarray Experiments

**Figure 1:**
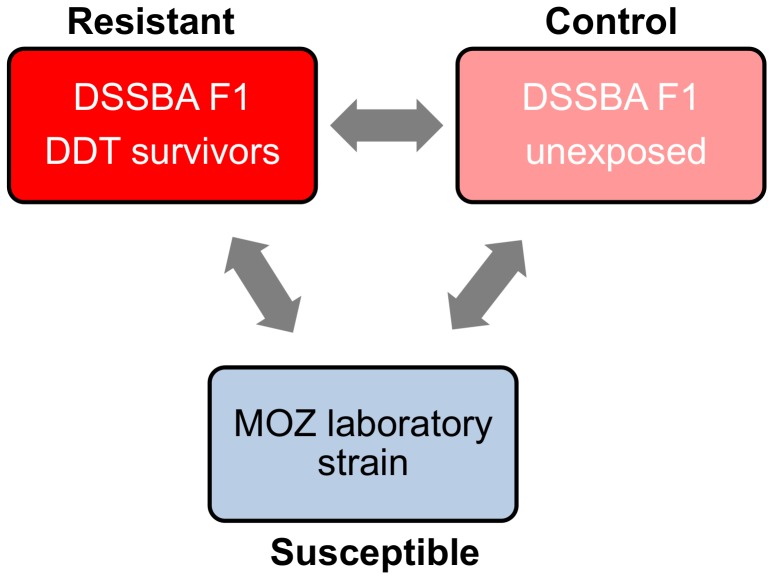
Microarray experimental design for investigating DDT resistance in *An. arabiensis* from a polluted breeding site in Dioulassoba. Gene expression profiles of mosquitoes from Dioulassoba exposed (DSSBA RES) and unexposed (DSSBA CON) to DDT (4%) were compared to a laboratory *An. arabiensis* strain (MOZ SUS).

A whole-genome microarray approach was used to determine whether DDT resistance in *An. arabiensis* is associated with differences in gene expression. The transcriptional profiles of three experimental treatments were compared against each other (Figure 1): (i) DSSBA RES - *An. arabiensis* from Dioulassoba surviving one-hour exposure to DDT (4%), (ii) DSSBA CON - *An. arabiensis* from Dioulassoba exposed to insecticide-free control papers and (iii) MOZ SUS – a laboratory colony of *An. arabiensis* originating from Mozambique. MOZ SUS is maintained at the Liverpool School of Tropical Medicine, is fully susceptible to all WHO-approved insecticides and was not exposed to DDT as part of this study.

**Table 1:** The genotypes at the 1014 *kdr* locus in *An. arabiensis* from Dioulassoba unexposed and exposed to DDT 4% in WHO susceptibility tests.

		Genotypes at the 1014 *kdr* locus					
Treatment	N	LL	LS	SS	f(1014L)	f(1014S)	HW χ^2^ *
Non-exposed	63	24	28	11	0.60	0.40	0.32
DDT 4% (1 hour)	76	22	42	12	0.57	0.43	1.18

* Hardy-Weinburg χ^2^ value calculated using online tool described by [38].

All mosquitoes used in the microarray were adult females, five to seven day-old (i.e preserved 48 hours post bioassay) identified as *An. arabiensis* by PCR. Total RNA was extracted from six replicate pools of eight individuals from the three adult *An. arabiensis* samples (DSSBA RES, DSSBA CON, MOZ SUS) using TRIzol® reagent (Invitrogen) following the manufacturer’s instructions. RNA was treated with two units of TURBO DNase™ (Ambion) to remove any remaining genomic DNA. The quantity and integrity of RNA was analysed using a NanoDrop (ND1000) Spectrophotometer (NanoDrop Technologies) and 2100 Bioanalyzer (Agilent technologies, Santa Clara, CA, USA). Two independent extractions were pooled to generate single biological replicates (a total of 16 mosquitoes per replicate). In total, three biological replicates per strain were used in the microarrays. cRNA was amplified from a starting template of 100 ng of total RNA and labeled with Cy3 and Cy5 fluorescent dyes using the two-color Low Input Quick Amp Labeling Kit (Agilent Technologies) according to the manufacturer’s instructions. Samples were column purified (Qiagen) and the Cy3/Cy5 specific activity and cRNA quantity measured using a spectrophotometer (NanoDrop Technologies) and Bioanalyzer (Agilent Technologies).

**Figure 2:**
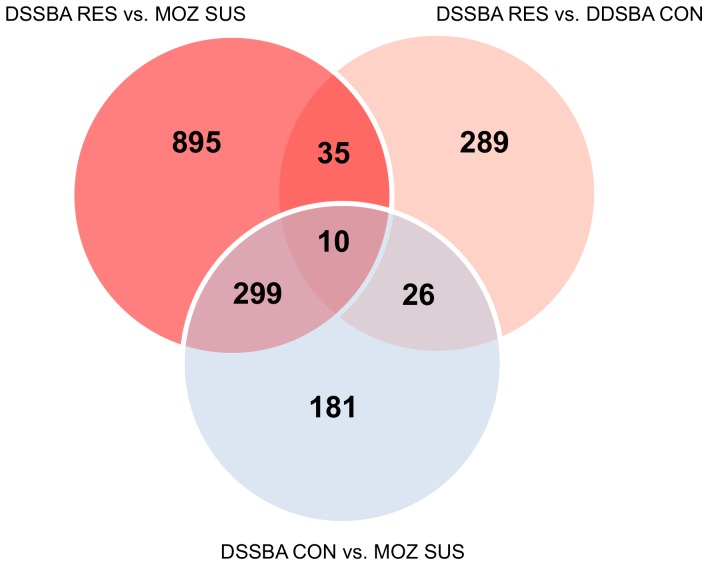
Venn diagram showing the pattern of differential expression among the microarray comparisons. Microarray probes were considered significantly expressed at p<0.05 after the application of a multiple testing correction (Benjamini-Hochberg).

**Table 2:** The most overrepresented biological terms associated with probes significantly differentially expressed in the microarray experiments comparing field *An. arabiensis* populations from Burkina Faso (DSSBA RES and DSSBA CON) and an insecticide-susceptible strain from Mozambique (MOZ SUS).

Microarray comparison	Category	Term	No. of probes	%	*p-value* Δ	Fold Enrichment
*DSSBA RES vs. MOZ SUS* *	GOTERM_MF_FAT	GO:0046906∼tetrapyrrole binding	27	2.72	0.00715	2.473
	GOTERM_MF_FAT	GO:0020037∼heme binding	27	2.72	0.00715	2.473
	SP_PIR_KEYWORDS	Iron	24	2.42	0.02218	2.346
	COG_ONTOLOGY	Secondary metabolites biosynthesis, transport, and catabolism	20	2.01	0.02298	2.186
	SP_PIR_KEYWORDS	Monooxygenase	17	1.71	0.02632	2.695
	GOTERM_MF_FAT	GO:0005506∼iron ion binding	33	3.32	0.02643	2.010
	SP_PIR_KEYWORDS	heme	19	1.91	0.03231	2.397
*DSSBA RES vs. DSSBA CON*	PIR_SUPERFAMILY	PIRSF000503:glutathione transferase	7	2.37	0.00007	14.163
	INTERPRO	IPR004045:Glutathione S-transferase, N-terminal	9	3.05	0.00023	11.649
	INTERPRO	IPR017933:Glutathione S-transferase/chloride channel, C-terminal	9	3.05	0.00025	10.590
	INTERPRO	IPR004046:Glutathione S-transferase, C-terminal	8	2.71	0.00082	10.712
	INTERPRO	IPR010987:Glutathione S-transferase, C-terminal-like	8	2.71	0.00100	10.021
*DSSBA CON vs. MOZ SUS* *	SP_PIR_KEYWORDS	oxidoreductase	26	6.30	3.28E−07	4.081
	SP_PIR_KEYWORDS	heme	18	4.36	5.30E−07	5.650
	SP_PIR_KEYWORDS	iron	19	4.60	3.22E−06	4.622
	SP_PIR_KEYWORDS	Monooxygenase	15	3.63	3.33E−06	5.917
	INTERPRO	IPR017973:Cytochrome P450, C-terminal region	17	4.12	3.41E−06	5.992
	INTERPRO	IPR017972:Cytochrome P450, conserved site	17	4.12	5.62E−06	6.069
	COG_ONTOLOGY	Secondary metabolites biosynthesis, transport, and catabolism	17	4.12	8.61E−06	4.096
	INTERPRO	IPR001128:Cytochrome P450	17	4.12	1.34E−05	5.319
	GOTERM_BP_FAT	GO:0055114∼oxidation reduction	31	7.51	1.54E−05	3.019
	GOTERM_MF_FAT	GO:0046906∼tetrapyrrole binding	19	4.60	0.0002	3.961
	GOTERM_MF_FAT	GO:0020037∼heme binding	19	4.60	0.0002	3.961
	INTERPRO	IPR002401:Cytochrome P450, E-class, group I	13	3.15	0.0010	5.028
	GOTERM_MF_FAT	GO:0005506∼iron ion binding	21	5.08	0.0027	2.911
	GOTERM_MF_FAT	GO:0009055∼electron carrier activity	19	4.60	0.0036	2.988
	INTERPRO	IPR013781:Glycoside hydrolase, subgroup, catalytic core	10	2.42	0.0069	5.460
	SP_PIR_KEYWORDS	metal-binding	29	7.02	0.0128	1.969
	INTERPRO	IPR016040:NAD(P)-binding domain	14	3.39	0.0258	3.276
	SP_PIR_KEYWORDS	glycosidase	5	1.21	0.0380	8.109
	GOTERM_MF_FAT	GO:0043169∼cation binding	68	16.46	0.0435	1.420
	GOTERM_MF_FAT	GO:0043167∼ion binding	68	16.46	0.0435	1.420
	INTERPRO	IPR002198:Short-chain dehydrogenase/reductase SDR	9	2.18	0.0478	4.557

* Functional annotation performed on genes commonly expressed in both DSSBA RES and DSSBA CON compared with MOZ SUS strain.

Δ *p*-value after Benjamini-Hochberg multiple testing correction.

Labeled cRNAs were hybridized to the Agilent 8×15 k ‘*Anopheles gambiae*’ array (AGAM_15K; ArrayExpress website. Available: http://www.ebi.ac.uk/arrayexpress. Accessed 2012 Sep 4) designed at the Liverpool School of Tropical Medicine [33]. The *An. gambiae* chip contains eight replicated arrays spotted with 14,071 probes for the 12,604 *An. gambiae s.s*. genes identified in the Ensembl P3.5 annotation (September 2009). On each array, 281 detoxification genes which were previously included on the *Anopheles gambiae* detox chip [40], are represented by four separate probes. Microarray comparisons were made between the three biological replicates of each strain with dye-swaps. Hybridizations were conducted for 17 hours at 65°C at 10 rpm rotation and washed following the manufacturer’s protocol (Agilent Technologies). Scanning of each microarray slide was performed with the Agilent G2565AA/G2565BA Microarray Scanner System using the Agilent Feature Extraction Software (Agilent Technologies).

**Table 3:** P450s significantly expressed between the Dioulassoba populations (DSSBA RES and DSSBA CON) and MOZ SUS.

		Microarray relative fold-change	
P450 Name	Ensembl Transcript ID	DSSBA RES vs. MOZ SUS	DSSBA CON vs. MOZ SUS
*CYP9J5*	AGAP012296-RA	7.72	7.09
*CYP6Z2*	AGAP008218-RA	6.12	7.48
*CYP6Z3*	AGAP008217-RA	5.76	4.50
*CYP6M3*	AGAP008213-RA	3.16	2.28
*CYP4H24*	AGAP000088-RA	2.83	2.90
*CYP12F1*	AGAP008022-RA	2.26	2.70
*CYP325C3*	AGAP009696-RA	0.19	0.12
*CYP325D1*	AGAP002206-RA	0.18	0.31
*CYP325C1*	AGAP002207-RA	0.15	0.08
*CYP325C2*	AGAP002205-RA	0.13	0.06

**Table 4:** Differentially expressed glutathione-S-transferases between DSSBA RES and DSSBA CON.

GST Name	Ensembl Transcript ID	Microarray relative fold-change
*GSTE6*	AGAP009191-RA	2.86
*GSTU4*	AGAP009190-RA	2.38
*GSTD1_5*	AGAP004164-RA	2.37
*GSTD3*	AGAP004382-RA	2.28
*GSTD11*	AGAP004378-RA	2.26
*GSTE5*	AGAP009192-RA	2.23
*GSTD10*	AGAP004383-RA	0.39

**Table 5:** The ten differentially expressed probes common in all three microarray comparisons.

		Microarray fold-change		
Gene Name	Ensembl Transcript ID	DSSBA RES vs.MOZ SUS	DSSBA CON vs.MOZ SUS	DSSBA RES vs.DSSBA CON
AGAP003646-PA [Anopheles gambiae str. PEST]	AGAP003646-RA	65.77	24.32	2.04
protease m1 zinc metalloprotease	AGAP004808-RA	0.47	0.13	3.01
alpha amylase	AGAP006371-RA	2.84	5.62	0.49
GSTD3	AGAP004382-RA	5.34	2.53	2.31
GSTD3	AGAP004382-RA	5.63	2.63	2.28
GSTD3	AGAP004382-RA	5.40	2.55	2.26
odorant-binding protein obpjj83a	AGAP009402-RA	3.23	16.28	0.14
odorant-binding protein	AGAP003530-RA	3.06	3.84	0.49
COEAE2G - Carboxylesterase	AGAP006723-RA	6.27	2.42	2.79
alkaline nuclease	AGAP011046-RA	6.05	19.63	0.24

**Figure 3:**
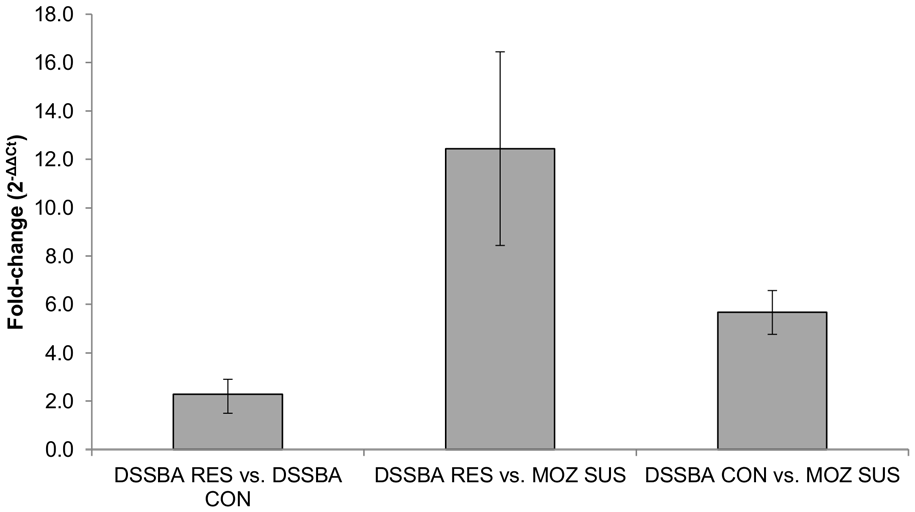
Fold-change in *GSTD3* expression for microarray comparisons using quantitative qPCR. Fold changes are expressed using the 2^−ΔΔCt^ formula with 95% C.I. [39].

All microarray analyses were performed in Genespring GX 11.5 software (Agilent technologies). Transcripts flagged as ‘detected’ or ‘non-detected’ in at least five out of six hybridizations were filtered from the probe set and from these, only transcripts with the background subtracted signal greater than the background standard deviation in at least five out of six hybridizations were taken forward for statistical analysis. The transcription ratios were subjected to a one sample Student’s t-test against the baseline value of 1 (equal gene expression in both strains) with a Benjamini-Hochberg FDR multiple testing correction. Transcripts with over a two-fold difference in expression and a t-test *p*-value below 0.05 after multiple testing were considered significantly different. The fold-changes of replicated probes were averaged for each representative gene. The microarray data have been deposited in ArrayExpress (ArrayExpress website. Available: http://www.ebi.ac.uk/arrayexpress/. Accessed 2012 Sep 4).

The transcript IDs of significantly up- or down-regulated genes were entered into the Functional Annotation tool available in the Database for Annotation Visualization and Integrated Discovery (DAVID) 6.7. DAVID systematically maps large lists of genes according to the associated biological annotation and calculates statistically significant enriched biological annotations from the target data set [41]. The most enriched biological terms were identified from each individual microarray comparison and presented according to their enrichment *p*-value (*p*<0.05) following Benjamini-Hochberg multiple testing.

### RT-qPCR Validation of Candidate Genes

Reverse-transcription quantitative PCR (RT-qPCR) was used to confirm the expression of two target genes selected from the microarray experiments; a glutathione-S-transferase, *GSTD3* (AGAP004382-RA) and an ABC transporter, *ABCG4* (AGAP001333-RA). Primers spanning exon-exon boundaries (to prevent gDNA amplification) were designed using PrimerBLAST (NCBI) to minimise off-target amplification. First-strand cDNA was reverse-transcribed from approximately 5 µg of total RNA using 200 units/µl of SuperScript™ III Reverse Transcriptase (Invitrogen) and 1 µl of oligo(dT)_20_ (50 µM), in the presence of 1 µl of RNaseOUT™ (Invitrogen). Samples were run on the Mx3005P QPCR system (Agilent Technologies) using a thermal profile of 95°C for 3 minutes followed by 40 cycles of 95°C for 10 seconds and 60°C for 10 seconds. qPCR reactions (20 µl) composed of 5 µl of cDNA (1 ng/µl), 1× Brilliant III Ultra-Fast SYBR® QPCR Master Mix (Agilent Technologies) and 300 nM of forward and reverse primers. Disassociation curves were run after each qPCR reaction to check for non-specific PCR products. The PCR efficiency and dynamic range of each primer set was determined using calibration curves generated from running qPCR reactions over five serial dilutions (five-fold) of input cDNA. Information on primers and qPCR efficiencies are given in Table S1. The same three biological replicates used for microarrays were analysed per treatment group and all qPCR reactions were performed in triplicate. No-template controls (NTCs) were run throughout all experiments. The expression of these genes was quantified using the comparative C_T_ method (otherwise known as the 2^−ΔΔCt^ method) [42]. Each target gene was normalised against the mean of two reference genes; ribosomal protein S7 (AGAP010592) and actin-5C (AGAP000651).

## Results

### DDT Resistance in *An. arabiensis* from Dioulassoba

To determine DDT resistance levels from the polluted site of Dioulassoba, a total of 313 and 262 female *An. gambiae s.l.* were exposed for one hour to DDT (4%) during the rainy (August-September, 2009) and dry season (January 2010), respectively. Mosquito mortality in the rainy season was 65.8% (N = 313; 95% CI = 60.3−70.1%) and in the dry season was 70.4% (N = 262 95% CI = 64.4−75.9%) with no significant difference observed (Fisher’s *p = *0.281). The mortality rates observed here indicate the presence of DDT resistance in *An. gambiae s.l.* from this urban area.

Sub-samples of *An. gambiae s.l.* from the control bioassays were PCR-identified to determine the frequency of different members of the species complex from Dioulassoba. In August-September 2009, *An. arabiensis* accounted for 0.790 (N = 77; 95% CI = 0.680−0.871) of *An. gambiae s.l.*, whereas this frequency significantly increased (χ^2^ *p* = 0.007) to 0.985 in January (N = 66; 95% CI = 0.909−0.999). *An. arabiensis* is therefore the dominant member of the *An. gambiae* complex in this area.

### Genotyping of kdr

The frequencies of the *kdr* alleles *1014F* and *1014S* in *An. arabiensis* from Dioulassoba were determined from a sub-sample of mosquitoes surviving (N = 63) and non-exposed (N = 76) to DDT using TaqMan® PCR (Table 1). The *1014F* mutation was not found in *An. arabiensis*. The frequency of *1014S* was high and remains in HW-equilibrium (*p*>0.05) but did not differ between non-exposed (0.40) and DDT survivors (0.43) (χ^2^ *p = *0.610). *1014F* was at a high frequency (0.88) from a small number of *An. gambiae s.s.* tested (exposed, N = 16; unexposed, N = 1), while *1014S* was not found.

### Transcription Profiles in *An. arabiensis* from Dioulassoba

All mosquito samples included in the microarrays were pre-identified as *An. arabiensis*. Gene expression profiles were compared between three groups of *An. arabiensis*; (i) mosquitoes from Dioulassoba surviving DDT exposure (DSSBA RES), (ii) mosquitoes from Dioulassoba unexposed to insecticide (DSSBA CON), (iii) a laboratory insecticide-susceptible strain of *An. arabiensis* (MOZ SUS) (Figure 1). This three-way experimental design enabled identification of genes differentially expressed in the DDT resistant proportion of the DSSBA population, by comparing to the global population with the same genetic and environmental background and, in addition, provided information on expression relative to a standard reference strain, important for cross population/cross country comparisons. As mosquitoes were stored 48 hours post DDT exposure, the induction effect of insecticide exposure was expected to be minimal and hence the primary objective was to compare constitutive changes in gene expression.

The number of significantly differentially expressed probes for each comparison is given in Figure 2. As expected, the greatest number of differentially expressed genes was observed between DSSBA RES/DSSBA CON and MOZ SUS. A total of 360 probes (2.6% of the total probes on the array) were differentially expressed between DSSBA RES and DSSBA CON. The full list of genes differentially expressed in each comparison is given in the Supporting Information (Tables S2, S3, S4).

Enrichment analysis was initially performed on probes commonly expressed in both DSSBA RES and DSSBA CON compared with MOZ SUS (N = 299). This revealed gene-terms consistent with increased monooxygenase activity (Table 2), suggesting constitutive differences in P450 activity between the resistant field mosquitoes and the susceptible laboratory strain. Thirty probes representing 10 individual P450s were significantly differentially expressed in DSSBA RES and DSSBA CON compared to MOZ SUS. Of these, six were commonly up-regulated (*CYP9J5*, *CYP6Z2*, *CYP6Z3*, *CYP6M3*, *CYP4H24*, *CYP12F1*) whereas only members of the *CYP325* class were down-regulated (Table 3). Moreover, a minimum of three probes were consistently up-regulated, representing *CYP9M1* (2.62-fold) and *CYP4C36* (3.66-fold), in DSSBA RES compared with DSSBA CON.

When the enrichment analysis was performed on probe sets differentially expressed between DSSBA RES and DSSBA CON, only gene-terms associated with glutathione-S-transferase (GST) activity were overrepresented and these possessed a high fold-enrichment score (>10) (Table 2). Thirteen probes representing seven GSTs were differentially expressed between DSSBA RES and DSSBA CON. Six of these GSTs were up-regulated in DSSBA RES: *GSTE6*, *GSTU4*, *GSTD1*, *GSTD3*, *GSTD11* and *GSTE5* (Table 4). Of these, *GSTD3* (AGAP004382) was significantly over-expressed in all microarray comparisons with a fold-change (average of three over-expressed probes) in the following descending order: DSSBA RES vs. MOZ SUS (fold-change = 5.46), DSSBA CON vs. MOZ SUS (fold-change = 2.57) and DSSBA RES vs. DSSBA CON (fold-change = 2.28) (Table 5).

Three genes from the ABC transporter family are expressed at higher levels in the DSSBA mosquitoes compared to MOZ SUS. All of these are members of the G family of half transporters which include the eye pigment precursors. One ABCG gene, *ABCG4* (AGAP001333) is found at 2-fold higher levels in DSSBA RES than DSSBA CON. The closest ortholog to this gene in humans is *ABCG2*, which has been associated with resistance to cancer drugs [43]. Expression of *ABCB1* is also increased in the DDT resistant subset relative to the non-exposed group. The ABCB family in vertebrates is also associated with multidrug resistance and contains the MDRR1 transporter [44]. A list of all ABC transporters expressed in microarray experiments are presented in Table S5.

Real-time qPCR confirmed the over-expression of *GSTD3* in each comparison (Figure 3), although, as reported previously for the Agilent array platform, expression ratios obtained from RT-qPCR were on the whole higher than those generated by the microarrays [28], [45]. RT-qPCR also confirmed the over expression of *ABCG4* (AGAP001333) in DSSBA RES compared with DSSBA CON (2^−ΔΔCt^ fold change = 4.97).

### Discussion

*Anopheles* breeding sites are not restricted to clearly defined habitats in urban ecosystems [8]. Members of the *An. gambiae* species complex are increasingly found ovipositing and breeding in atypical polluted or domestic water bodies [12]. Our study site (Dioulassoba) represents one such area where mosquitoes breed within and alongside a permanent stream heavily polluted with organic and domestic waste. *An. arabiensis* comprised 0.79 (rainy season) and 0.99 (dry season) of *An. gambiae s.l.* collected from Dioulassoba, confirming previous findings which show that this species has become the predominant member of the *An. gambiae* complex in this site over the past decade [35]. A similar pattern is emerging in Burkina Faso’s capital Ouagadougou [9] and other areas of West Africa [46].

Insecticide resistance is an emerging threat for LLIN and IRS efficacy in rural areas but much less attention has focussed on the urban setting. Although control of urban malaria vectors may involve targeting the immature stages via measures such as larviciding and environmental management, prevention of transmission via the use of LLINs is usually the primary control measure. Unplanned development of towns and cities often leads to an increase in the number of available breeding sites, many of which will contain higher levels of pollutants or other xenobiotics than the typically more pristine rural sites. Mosquito larvae exposed to anthropogenic chemicals have been shown to be better able to survive insecticide exposure in laboratory settings [47]. The use of pesticides in urban agriculture, and the exposure to petroleum products have both been identified as a potentially strong driving force behind the selection of resistance [48], [49].

Resistance to DDT and permethrin was reported in Bobo-Dioulasso over 10 years ago but *An. arabiensis* only comprised 0–8.3% of the *An. gambiae s.l.* population at this time [34]. In bioassays conducted in 2009–2010 a high prevalence of DDT resistance (mortality of 65.8–70.4%) was detected in *An. arabiensis* in the city. At the same time, resistance to DDT was observed in *An. arabiensis* from four rural sentinel sites (mortality after diagnostic dose exposure ranging from 78.9–84.2% [50]). Thus resistance is slightly more prevalent in the urban setting. More striking, however, is the difference in the frequency of the *1014S* kdr mutation between the urban and rural sites.

The *1014S* allele frequency is 40–43% in *An. arabiensis* from Dioulassoba. Frequencies of only 6–11% for *1014S* have been observed in *An. arabiensis* from the four rural sites in Burkina Faso in the same time period [50]. The *1014S* mutation has been detected in *An. arabiensis* from Sudan [51], Uganda [52], Kenya [53] and Benin [54] but in each case, the reported frequencies are low. Although no dead mosquitoes were screened from the bioassays to establish a genotype-phenotype link, previous field data suggests that *1014S* is associated with higher levels of DDT and lower levels of permethrin resistance [52], [55], [56]. *In vitro* expression work using modified *Drosophila* VGSCs in *Xenopus* oocytes has shown that the *1014S* allele confers higher resistance to DDT than the *1014F* variant [57]. Clearly, the high frequency of *1014S* is indicative of a selective benefit for this mutation though whether this benefit is a consequence of improved survival to insecticide exposure remains unknown. Since the frequency of the *1014S* allele was only 40% in the resistant population, this suggests that other resistant mechanisms are present.

We applied whole-genome microarrays to identify additional putative DDT resistance mechanisms in *An. arabiensis*. Although the microarray used here is designed for studying transcription in *An. gambiae s.s.*, the high degree of homology between the sibling species permitted cross-species hybridisation [58], [59]. In this study, we used a three-way comparison between field mosquitoes surviving (DSSBA RES) or non-exposed (DSSBA CON) to DDT with a laboratory susceptible strain (MOZ-SUS) to assess constitutive gene expression. Several probes were commonly transcribed in both DSSBA RES and DSSBA CON compared with MOZ-SUS (N = 299). The most over-represented biological terms from this gene list were consistent with P450 activity. It is possible that fast-evolving genes such as P450s have diverged sufficiently between *An. gambiae s.s.* and *An. arabiensis* to augment issues of cross hybridisation that are common to all microarray studies on large gene families and further qPCR analysis is needed to confirm the expression patterns observed. It is of interest that two of the six P450s up-regulated in *An. arabiensis* from Dioulassoba (*CYP6Z2* and *CYP12F1*) were over-transcribed in the DDT-resistant *An. gambiae s.s.* strain ZAN/U [40]. The over-transcription of P450 genes and their association with insecticide resistance has been well documented in insect pests of agriculture and human disease vectors [27], [28], [60].

In DSSBA RES, genes with putative glutathione transferase (GST) activity were enriched amongst the differentially expressed subset. Increased GST activity is known to confer DDT resistance in mosquitoes [61], [62] by catalysing the removal of a chlorine group from the pesticide. Interestingly, *GSTD3* was over-expressed in all three microarray comparisons (and confirmed by RT-qPCR). Delta class GSTs have been implicated in insecticide resistance [63] but their role has previously thought to be relatively minor compared with those from the epsilon class (for example *GSTE2*). Functional validation of the interaction between *GSTD3* and DDT is required to understand the relevance of the over-expression of this gene in all microarray comparisons.

Finally, members of the ABC-transporter family (otherwise known as P-glycoproteins) were over-expressed in *An. arabiensis* from Dioulassoba. ABC-transporters pump foreign molecules out of insect cells using an ATP-dependent mechanism. ABC-transporters are increasingly implicated in pesticide (herbicide, fungicide and insecticide) resistance although the precise mechanism is not understood [64], [65]. The over-expression of an ABC-transporter (*ABCB4*) through gene amplification has recently been reported from pyrethroid-resistant *Aedes aegypti* [66].

Insecticide resistance is well established in *Anopheles* vectors throughout Africa and presents a genuine threat to malaria control. Until recently, *An. arabiensis* was generally regarded as the more susceptible sibling species compared to sympatric populations of *An. gambiae s.s.* in Burkina Faso and nearby regions of West Africa - this is not the case in Bobo-Dioulasso. The precise factors driving the selection for DDT resistance in *An. arabiensis* from Dioulassoba remain to be elucidated but an increase in insecticide-based interventions (including personal sprays and coils) and exposure to organic and anthropogenic pollutants may be important. Understanding the selection pressures in urban environments is important given the rapid rate of urbanisation, and concomitant increasing role of urban malaria in the epidemiology of this disease.

## Supporting Information

Table S1 Quantitative PCR (qPCR) primers for target genes selected from microarrays.
(XLSX)

Table S2 List of differentially expressed *An. arabiensis* transcripts from DSSBA RES versus MOZ microarray.
(XLSX)

Table S3 List of differentially expressed *An. arabiensis* transcripts from DSSBA RES versus DSSBA CON microarray.
(XLSX)

Table S4 List of differentially expressed *An. arabiensis* transcripts from DSSBA CON versus MOZ microarray.
(XLSX)

Table S5 The ABC-transporters differentially expressed in all microarray comparisons.
(XLSX)

## References

[1] World Urbanization Prospects: the 2011 Revision [http://esa.un.org/unpd/wup/unup/index_panel1.html] Accessed September 4, 2012.

[2] Hay SI, Guerra CA, Tatem AJ, Atkinson PM, Snow RW (2005) Urbanization, malaria transmission and disease burden in Africa. Nat Rev Microbiol 3(1): 81–90. 15608702 10.1038/nrmicro1069 PMC3130901

[3] Keiser J, Utzinger J, De Castro MC, Smith TA, Tanner M, et al (2004) Urbanization in sub-Saharan Africa and implication for malaria control. Am J of Trop Med Hyg 71(2): 118–127. 15331827

[4] Donnelly MJ, McCall PJ, Lengeler C, Bates I, D’Alessandro U, et al (2005) Malaria and urbanization in sub-Saharan Africa. Malar J 4: 12. 15720713 10.1186/1475-2875-4-12 PMC552321

[5] Coetzee M, Craig M, le Sueur D (2000) Distribution of African malaria mosquitoes belonging to the *Anopheles gambiae* complex. Parasitol Today 16(2): 74–77. 10652493 10.1016/s0169-4758(99)01563-x

[6] Coluzzi M, Sabatini A, Petrarca V, Dideco MA (1979) Chromosomal differentiation and adaptation to human environments in the *Anopheles gambiae* complex. Trans R Soc Trop Med Hyg 73(5): 483–497. 394408 10.1016/0035-9203(79)90036-1

[7] Kerah-Hinzoumbé C, Peka M, Antonio-Nkondjio C, Donan-Gouni I, Awono-Ambene P, et al (2009) Malaria vectors and transmission dynamics in Goulmoun, a rural city in south-western Chad. BMC Infect Dis 9: 71. 19463189 10.1186/1471-2334-9-71 PMC2697161

[8] Sattler MA, Mtasiwa D, Kiama M, Premji Z, Tanner M, et al (2005) Habitat characterization and spatial distribution of *Anopheles* sp. mosquito larvae in Dar es Salaam (Tanzania) during an extended dry period. Malar J 4: 4. 15649333 10.1186/1475-2875-4-4 PMC546229

[9] Fournet F, Cussac M, Ouari A, Meyer PE, Toé HK, et al (2010) Diversity in anopheline larval habitats and adult composition during the dry and wet seasons in Ouagadougou (Burkina Faso). Malar J 2010 9: 78. 10.1186/1475-2875-9-78 PMC2907872 20298619

[10] Kristan M, Fleischmann H, della Torre A, Stich A, Curtis CF (2003) Pyrethroid resistance/susceptibility and differential urban/rural distribution of *Anopheles arabiensis* and *An. gambiae s.s.* malaria vectors in Nigeria and Ghana. Med Vet Ent 17(3): 326–332. 10.1046/j.1365-2915.2003.00449.x 12941018

[11] della Torre A, Tu ZJ, Petrarca V (2005) On the distribution and genetic differentiation of *Anopheles gambiae s.s.* molecular forms. Insect Biochem Molec Biol 35(7): 755–769. 15894192 10.1016/j.ibmb.2005.02.006

[12] Chinery WA (1984) Effects of ecological changes on the malaria vectors *Anopheles funestus* and the *Anopheles gambiae* complex of mosquitoes in Accra, Ghana. J Trop Med Hyg 87(2): 75–81. 6748132

[13] Chouaïbou M, Etang J, Brévault T, Nwane P, Hinzoumbé CK, et al (2008) Dynamics of insecticide resistance in the malaria vector *Anopheles gambiae s.l.* from an area of extensive cotton cultivation in Northern Cameroon. Trop Med Int Health 13(4): 476–486. 18248566 10.1111/j.1365-3156.2008.02025.x

[14] World Malaria Report (2010) World Health Organisation.

[15] Ranson H, N’Guessan R, Lines J, Moiroux N, Nkuni Z, et al (2011) Pyrethroid resistance in African anopheline mosquitoes: what are the implications for malaria control? Trends Parasitol 27(2): 91–98. 20843745 10.1016/j.pt.2010.08.004

[16] van den Berg H, Zaim M, Yadav RS, Soares A, Ameneshewa B, et al (2012) Global trends in the use of insecticides for vector-borne diseases. Environ Health Perspect 120(4): 577–582. 22251458 10.1289/ehp.1104340 PMC3339467

[17] Alou LPA, Koffi AA, Adja MA, Tia E, Kouassi PK, et al (2010) Distribution of ace-1^R^ and resistance to carbamates and organophosphates in *Anopheles gambiae s.s.* populations from Côte d’Ivoire. Malar J 9: 167. 20553593 10.1186/1475-2875-9-167 PMC2908637

[18] Dabiré KR, Diabate A, Namountougou M, Toé KH, Ouari A, et al (2009) Distribution of pyrethroid and DDT resistance and the L1014F kdr mutation in *Anopheles gambiae s.l.* from Burkina Faso (West Africa). Trans R Soc Trop Med Hyg 103(11): 1113–1120. 19246066 10.1016/j.trstmh.2009.01.008

[19] Yewhalaw D, Wassie F, Steurbaut W, Spanoghe P, Van Bortel (2011) Multiple Insecticide Resistance: An Impediment to Insecticide-Based Malaria Vector Control Program. PLoS ONE 6(1): 10.1371/journal.pone.0016066. 10.1371/journal.pone.0016066 PMC3020220 21264325

[20] Davies TGE, Field LM, Usherwood PNR, Williamson MS (2007) DDT, pyrethrins, pyrethroids and insect sodium channels. IUBMB Life 59(3): 151–162. 17487686 10.1080/15216540701352042

[21] Donnelly MJ, Corbel V, Weetman D, Wilding CS, Williamson MS, et al (2009) Does kdr genotype predict insecticide-resistance phenotype in mosquitoes? Trends Parasitol. 25(5): 213–219. 10.1016/j.pt.2009.02.007 19369117

[22] Jones CM, Liyanapathirana M, Agossa FR, Weetman D, Ranson H, et al (2012) Footprints of positive selection associated with a mutation (N1575Y) in the voltage-gated sodium channel of *Anopheles gambiae* . Proc Natl Acad Sci USA 109(17): 6614–6619. 22493253 10.1073/pnas.1201475109 PMC3340067

[23] Lynd A, Weetman D, Barbosa S, Yawson AE, Mitchell S, et al (2010) Field, Genetic, and Modeling Approaches Show Strong Positive Selection Acting upon an Insecticide Resistance Mutation in *Anopheles gambiae s.s.* . Mol Biol Evol 27(5): 1117–1125. 20056691 10.1093/molbev/msq002 PMC2877529

[24] Diabaté A, Baldet T, Chandre E, Dabiré KR, Simard F, et al (2004) First report of a kdr mutation in *Anopheles arabiensis* from Burkina Faso, West Africa. J Am Mosq Control Assoc 20(2): 195–196. 15264630

[25] Djégbé I, Boussari O, Sidick A, Martin T, Ranson H, et al (2011) Dynamics of insecticide resistance in malaria vectors in Benin: first evidence of the presence of L1014S kdr mutation in *Anopheles gambiae* from West Africa. Malar J 10: 261. 21910856 10.1186/1475-2875-10-261 PMC3179749

[26] Santolamazza F, Calzetta M, Etang J, Barrese E, Dia I, et al (2008) Distribution of knock-down resistance mutations in *Anopheles gambiae* molecular forms in west and west-central Africa. Malar J 7: 74. 18445265 10.1186/1475-2875-7-74 PMC2405802

[27] Daborn PJ, Yen JL, Bogwitz MR, Le Goff G, Feil E, et al (2002) A single P450 allele associated with insecticide resistance in *Drosophila* . Science 297(5590): 2253–2256. 12351787 10.1126/science.1074170

[28] Puinean AM, Foster SP, Oliphant L, Denholm I, Field LM, et al (2010) Amplification of a Cytochrome P450 Gene Is Associated with Resistance to Neonicotinoid Insecticides in the Aphid *Myzus persicae* . PLoS Genetics 6(6): 10.1371/journal.pgen.1000999. 10.1371/journal.pgen.1000999 PMC2891718 20585623

[29] Muller P, Warr E, Stevenson BJ, Pignatelli PM, Morgan JC, et al (2008) Field-Caught Permethrin-Resistant *Anopheles gambiae* Overexpress CYP6P3, a P450 That Metabolises Pyrethroids. PLoS Genetics 2008 4(11): 10.1371/journal.pgen.1000286. 10.1371/journal.pgen.1000286 PMC2583951 19043575

[30] Chiu TL, Wen ZM, Rupasinghe SG, Schuler MA (2008) Comparative molecular modeling of *Anopheles gambiae* CYP6Z1, a mosquito P450 capable of metabolizing DDT. Proc Natl Acad Sci USA 105(26): 8855–8860. 18577597 10.1073/pnas.0709249105 PMC2449330

[31] Stevenson BJ, Bibby J, Pignatelli P, Muangnoicharoen S, O’Neill PM, et al (2011) Cytochrome P450 6M2 from the malaria vector *Anopheles gambiae* metabolizes pyrethroids: Sequential metabolism of deltamethrin revealed. Insect Biochem Molec Biol 41(7): 492–502. 21324359 10.1016/j.ibmb.2011.02.003

[32] Ranson H, Rossiter L, Ortelli F, Jensen B, Wang XL, et al (2001) Identification of a novel class of insect glutathione S-transferases involved in resistance to DDT in the malaria vector *Anopheles gambiae* . Biochem J 359: 295–304. 11583575 10.1042/0264-6021:3590295 PMC1222147

[33] Mitchell SN, Stevenson BJ, Müller P, Wilding CS, Egyir-Yawson A, et al (2012) Identification and validation of a gene causing cross-resistance between insecticide classes in *Anopheles gambiae* from Ghana. Proc Natl Acad Sci 109(16): 6147–6152. 22460795 10.1073/pnas.1203452109 PMC3341073

[34] Diabaté A, Baldet T, Chandre F, Akogbeto M, Guiguemde TR, et al (2002) The role of agricultural use of insecticides in resistance to pyrethroids in *Anopheles gambiae* s.l. in Burkina Faso. Am J Trop Med Hyg 67(6): 617–622. 12518852 10.4269/ajtmh.2002.67.617

[35] Djogbenou L, Dabiré R, Diabaté A, Kengne P, Akogbeto M, et al (2008) Identification and geographic distribution of the ace-1^R^ mutation in the malaria vector *Anopheles gambiae* in south-western Burkina Faso, West Africa. Am J Trop Med Hyg 78(2): 298–302. 18256433

[36] World Health Organisation (1998) Test procedures for insecticide resistance monitoring in malaria vectors, bio-efficacy and persistence of insecticides on treated surfaces.

[37] Scott JA, Brogdon WG, Collins FH (1993) Identification of single specimens of the *Anopheles gambiae* complex by the polymerase chain reaction. Am J Trop Med Hyg 49(4): 520–529. 8214283 10.4269/ajtmh.1993.49.520

[38] Bass C, Nikou D, Donnelly MJ, Williamson MS, Ranson H, et al (2007) Detection of knockdown resistance (kdr) mutations in *Anopheles gambiae*: a comparison of two new high-throughput assays with existing methods. Malar J 6: 111. 17697325 10.1186/1475-2875-6-111 PMC1971715

[39] Rodriguez S, Gaunt TR, Day INM (2009) Hardy-Weinberg Equilibrium Testing of Biological Ascertainment for Mendelian Randomization Studies. Am J Epidemiol 169(4): 505–514. 19126586 10.1093/aje/kwn359 PMC2640163

[40] David JP, Strode C, Vontas J, Nikou D, Vaughan A, et al (2005) The *Anopheles gambiae* detoxification chip: A highly specific microarray to study metabolic-based insecticide resistance in malaria vectors. Proc Natl Acad Sci USA 102(11): 4080–4084. 15753317 10.1073/pnas.0409348102 PMC554807

[41] Huang DW, Sherman BT, Lempicki RA (2009) Systematic and integrative analysis of large gene lists using DAVID bioinformatics resources. Nat Protoc 4(1): 44–57. 19131956 10.1038/nprot.2008.211

[42] Schmittgen TD, Livak KJ (2008) Analyzing real-time PCR data by the comparative CT method. Nat Protoc 3(6): 1101–1108. 18546601 10.1038/nprot.2008.73

[43] Tarr PT, Tarling EJ, Bojanic DD, Edwards PA, Baldan A (2009) Emerging new paradigms for ABCG transporters. BBA-Mol Cell Biol L 1791(7): 584–593. 10.1016/j.bbalip.2009.01.007 PMC2698934 19416657

[44] Sarkadi B, Homolya L, Szakacs G, Varadi A (2006) Human multidrug resistance ABCB and ABCG transporters: Participation in a chemoimmunity defense system. Physiol Rev 86(4): 1179–1236. 17015488 10.1152/physrev.00037.2005

[45] Marcombe S, Poupardin R, Darriet F, Reynaud S, Bonnet J, et al. (2009) Exploring the molecular basis of insecticide resistance in the dengue vector *Aedes aegypti*: a case study in Martinique Island (French West Indies). BMC Genomics 2009 10; 494. 10.1186/1471-2164-10-494 PMC2770535 19857255

[46] Onyabe D, Conn JE (2001) The Distribution of Two Major Malaria Vectors, *Anopheles gambiae* and *Anopheles arabiensis*, in Nigeria. Mem Inst Oswaldo Cruz, Rio de Janeiro 2001 98(8): 1081–1084. 10.1590/s0074-02762001000800009 11784926

[47] Poupardin R, Riaz MA, Jones CM, Chandor-Proust A, Reynaud S, et al (2012) Do pollutants affect insecticide-driven gene selection in mosquitoes? Experimental evidence from transcriptomics. Aquat Toxicol 114–115: 49–57. 10.1016/j.aquatox.2012.02.001 22406618

[48] Antonio-Nkondjio C, Fossog BT, Ndo C, Djantio BM, Togouet SZ, et al (2011) *Anopheles gambiae* distribution and insecticide resistance in the cities of Douala and Yaounde (Cameroon): influence of urban agriculture and pollution. Malar J 10: 154. 21651761 10.1186/1475-2875-10-154 PMC3118161

[49] Dongus S, Nyika D, Kannady K, Mtasiwa D, Mshinda H, et al (2009) Urban agriculture and *Anopheles* habitats in Dar es Salaam, Tanzania. Geospatial Health 3(2): 189–210. 19440962 10.4081/gh.2009.220

[50] Badolo A, Traore A, Jones CM, Sanou A, Flood L, et al (2012) Three years of insecticide resistance monitoring in *Anopheles gambiae* from Burkina Faso: resistance on the rise? Malar J 11: 232. 22799568 10.1186/1475-2875-11-232 PMC3489511

[51] Himeidan YE, Chen H, Chandre F, Donnelly MJ, Yan G (2007) Permethrin and DDT resistance in the malaria vector *Anopheles arabiensis* from eastern Sudan. Am J Trop Medicine Hyg 77(6): 1066–1068. 18165523

[52] Ramphul U, Boase T, Bass C, Okedi LM, Donnelly MJ, et al (2009) Insecticide resistance and its association with target-site mutations in natural populations of *Anopheles gambiae* from eastern Uganda. Trans R Soc Trop Med and Hyg 103(11): 1121–1126. 19303125 10.1016/j.trstmh.2009.02.014

[53] Stump AD, Atieli FK, Vulule JM, Besansky NJ (2004) Dynamics of the pyrethroid knockdown resistance allele in western Kenyan populations of *Anopheles gambiae* in response to insecticide-treated bed net trials. Am J Trop Med Hyg 70(6): 591–596. 15210997

[54] Djogbenou L, Pasteur N, Akogbeto M, Weill M, Chandre F (2011) Insecticide resistance in the *Anopheles gambiae* complex in Benin: a nationwide survey. Med Vet Ent 25(3): 256–267. 10.1111/j.1365-2915.2010.00925.x 21155858

[55] Ranson H, Jensen B, Vulule JM, Wang X, Hemingway J, et al (2000) Identification of a point mutation in the voltage-gated sodium channel gene of Kenyan *Anopheles gambiae* associated with resistance to DDT and pyrethroids. Insect Mol Biol 9(5): 491–497. 11029667 10.1046/j.1365-2583.2000.00209.x

[56] Protopopoff N, Verhaeghen K, Van Bortel W, Roelants P, Marcotty T, et al (2008) A significant increase in kdr in *Anopheles gambiae* is associated with an intensive vector control intervention in Burundi highlands. Trop Med Int Health 13(12): 1479–1487. 18983277 10.1111/j.1365-3156.2008.02164.x

[57] Burton MJ, Mellor IR, Duce IR, Davies TGE, Field LM, et al (2011) Differential resistance of insect sodium channels with kdr mutations to deltamethrin, permethrin and DDT. Insect Biochem Mol Biol 41(9): 723–732. 21640822 10.1016/j.ibmb.2011.05.004

[58] Vontas J, David JP, Nikou D, Hemingway J, Christophides GK, et al (2007) Transcriptional analysis of insecticide resistance in *Anopheles stephensi* using cross-species microarray hybridization. Insect Mol Biol 16(3): 315–324. 17433071 10.1111/j.1365-2583.2007.00728.x

[59] Muller P, Chouaibou M, Pignatelli P, Etang J, Walker ED, et al (2008) Pyrethroid tolerance is associated with elevated expression of antioxidants and agricultural practice in *Anopheles arabiensis* sampled from an area of cotton fields in Northern Cameroon. Mol Ecol 17(4): 1145–1155. 18179425 10.1111/j.1365-294X.2007.03617.x

[60] Karunker I, Benting J, Lueke B, Ponge T, Nauen R, et al (2008) Over-expression of cytochrome P450 CYP6CM1 is associated with high resistance to imidacloprid in the B and Q biotypes of *Bemisia tabaci* (Hemiptera : Aleyrodidae). Insect Biochem Mol Biol 38(6): 634–644. 18510975 10.1016/j.ibmb.2008.03.008

[61] Prapanthadara LA, Hemingway J, Ketterman AJ (1993) Partial purification and characterisation of glutathione s-transferases involved in DDT resistance from the mosquito *Anopheles gambiae* . Pest Biochem Physiol 47(2): 119–133.

[62] Grant DF, Dietze EC, Hammock BD (1991) Glutathione-S-transferase isozymes in *Aedes aegypti* - purification, characterization, and isozyme-specific regulation. Insect Biochem 21(4): 421–433.

[63] Tang AH, Tu CPD (1994) Biochemical characterization of *Drosophila* glutathione S-transferases D1 and D21. J Biol Chem 269(45): 27876–27884. 7961718

[64] Gahan LJ, Pauchet Y, Vogel H, Heckel DG: An ABC Transporter Mutation Is Correlated with Insect Resistance to *Bacillus thuringiensis* Cry1Ac Toxin. PLoS Genetics 6(12): 10.1371/journal.pgen.1001248. 10.1371/journal.pgen.1001248 PMC3002984 21187898

[65] Buss DS, Callaghan A (2008) Interaction of pesticides with p-glycoprotein and other ABC proteins: A survey of the possible importance to insecticide, herbicide and fungicide resistance. Pest Biochem Physiol 90(3): 141–153.

[66] Bariami V, Jones CM, Poupardin R, Vontas J, Ranson H (2012) Gene Amplification, ABC Transporters and Cytochrome P450s: Unraveling the Molecular Basis of Pyrethroid Resistance in the Dengue Vector, *Aedes aegypti* . PLoS Negl Trop Dis 6(6): e1692. 22720108 10.1371/journal.pntd.0001692 PMC3373657

